# Molecular epidemiology of *Klebsiella pneumoniae* invasive infections over a decade at Kilifi County Hospital in Kenya

**DOI:** 10.1016/j.ijmm.2017.07.006

**Published:** 2017-10

**Authors:** Sonal P. Henson, Christine J. Boinett, Matthew J. Ellington, Ngure Kagia, Salim Mwarumba, Sammy Nyongesa, Neema Mturi, Samuel Kariuki, J. Anthony G. Scott, Nicholas R. Thomson, Susan C. Morpeth

**Affiliations:** a KEMRI-Wellcome Trust Research Programme, Kilifi, Kenya; b Centre for Tropical Medicine and Global Health, Nuffield Department of Clinical Medicine, Oxford University, Oxford, United Kingdom; c Wellcome Trust Sanger Institute, Hinxton, Cambridge, United Kingdom; d Public Health England, Microbiology Services Division, Addenbrooke's Hospital, Hills Road, Cambridge CB2 0QW, United Kingdom; e Centre for Microbiology Research, Kenya Medical Research Institute, Nairobi, Kenya; f London School of Hygiene and Tropical Medicine, Keppel St, London, United Kingdom

**Keywords:** MDR, multi drug resistance, ESBL, extended spectrum β-lactamase, KCH, Kilifi County Hospital, NDM-1, New Delhi Metallo-β-lactamase-1, MLST, multi locus sequence type, AMR, antimicrobial resistance, MGE, mobile genetic elements, *Klebsiella pneumoniae*, ESBL, Molecular epidemiology, Hospital-acquired infections, Community-acquired infections, Carbapenem

## Abstract

Multidrug resistant (MDR) *Klebsiella pneumoniae* is a common cause of nosocomial infections worldwide. Recent years have seen an explosion of resistance to extended-spectrum β-lactamases (ESBLs) and emergence of carbapenem resistance. Here, we examine 198 invasive *K. pneumoniae* isolates collected from over a decade in Kilifi County Hospital (KCH) in Kenya. We observe a significant increase in MDR *K. pneumoniae* isolates, particularly to third generation cephalosporins conferred by ESBLs. Using whole-genome sequences, we describe the population structure and the distribution of antimicrobial resistance genes within it. More than half of the isolates examined in this study were ESBL-positive, encoding CTX-M-15, SHV-2, SHV-12 and SHV-27, and 79% were MDR conferring resistance to at least three antimicrobial classes. Although no isolates in our dataset were found to be resistant to carbapenems we did find a plasmid with the genetic architecture of a known New Delhi metallo-β-lactamase-1 (NDM)-carrying plasmid in 25 isolates. In the absence of carbapenem use in KCH and because of the instability of the NDM-1 gene in the plasmid, the NDM-1 gene has been lost in these isolates. Our data suggests that isolates that encode NDM-1 could be present in the population; should carbapenems be introduced as treatment in public hospitals in Kenya, resistance is likely to ensue rapidly.

## 1 Introduction

*Klebsiella pneumoniae* is one of the top three bacteria of international concern in the 2014 WHO report on the global status of antibacterial resistance (WHO, 2014). At Kilifi County Hospital (KCH), *K. pneumoniae* caused 20% of total nosocomial bacteraemia episodes between 2002 and 2009 (Aiken et al., 2011). *K. pneumoniae* is prominently associated with nosocomial infections (Murphy et al., 1994) particularly in vulnerable individuals such as neonates, the elderly, or the chronically ill. As a part of the normal flora of the gastrointestinal tract it is also a common cause of community-acquired urinary tract infection worldwide.

*K. pneumoniae* are intrinsically resistant to penicillins such as ampicillin but typically susceptible to third generation cephalosporins such as ceftriaxone, an antibiotic in common use in hospitals around the world, including as empiric therapy for sepsis or meningitis in many African hospitals. Acquired resistance to third generation cephalosporins is mainly due to Extended Spectrum β-Lactamases (ESBLs) (Bradford, 2001), which have spread globally since the first identification of an ESBL gene in Germany in 1982 (Knothe et al., 1983). *Enterobacteriaceae* such as *K. pneumoniae*, carrying ESBL genes, are often multiply resistant to other commonly used antimicrobials including aminoglycosides and fluoroquinolones. In sub-Saharan African hospitals, including those in Kenya, enteric bacteria carrying ESBL genes have become a significant problem (Breurec et al., 2012, Kariuki et al., 2001, Mshana et al., 2009, Musoke and Revathi, 2000). In KCH the most common ESBL-carrying bacteria are *K. pneumoniae*, and outbreaks of invasive infection with ESBL-*K. pneumoniae* have been clinically noted to occur. The standard empiric treatment for suspected severe paediatric bacterial infections follows the Kenyan National and WHO guidelines (World Health Organization, 2014) of penicillin or ampicillin with gentamicin as first-line treatment. Ceftriaxone is used as second-line therapy, for those not responding to initial treatment after 48 h, or as first-line therapy for suspected bacterial meningitis outside of the neonatal period. ESBL-carrying *K. pneumoniae* isolates are usually resistant to both these empiric treatment regimens.

In high-income countries, carbapenems have been the treatment of choice for infections caused by ESBL-carrying bacteria (Perez and Bonomo, 2015), but they are often unavailable in the public health system in resource-poor settings. Resistance to carbapenems among *K. pneumoniae* is emerging across the globe (Johnson and Woodford, 2013); these infections can only be treated with last-resort drugs such as tigecycline or colistin, which are less effective, more toxic, and not widely available. Very little data on carbapenem-resistant *Enterobacteriaceae* are available from low income countries, especially in sub-Saharan Africa (WHO, 2014). Poirel and colleagues found NDM-1 producing isolates from a tertiary referral hospital in Nairobi, Kenya, where carbapenems are more readily available (Poirel et al., 2011b). At KCH seven cases of carbapenem-resistant *K. pneumoniae* have been identified since 2014.

Using whole genome sequencing methods, we describe the genetic diversity and the antimicrobial resistance genes (focusing on ESBLs) found in *K. pneumoniae* strains causing invasive disease over a decade in KCH, a primary government hospital, in rural Kenya.

## 2 Methods

### 2.1 Study site and samples

The study was conducted at Kilifi County Hospital (KCH) in rural Kilifi, one of Kenya’s poorest counties, where a majority of the population are rural farmers. Kilifi is 60 km from Mombasa, the nearest city with a secondary government hospital and several private secondary hospitals. KCH is a primary government hospital that serves a population of about one million. The KEMRI-Wellcome Trust Research Programme (KWTRP) has carried out surveillance of invasive bacterial infections at KCH for paediatrics since 1994 and for adults since 2007 (Scott et al., 2012).

At KCH, blood samples are routinely collected for culture for consenting paediatric and adult medical patients at admission (excluding accidents, fresh burns or snake bite). Cerebrospinal fluid and other sterile site samples are collected and cultured on admission if clinically indicated and, likewise, throughout the course of the illness subsequent specimens are collected for culture at the discretion of the attending clinician.

For the purpose of this analysis duplicate isolates from the same patient were not included. Blood cultures were performed using the automated BACTEC system (BD, USA) and isolates were identified using standard microbiological techniques (API, BioMerieux, France). Community-acquired infection was defined as culture-positive samples collected upon admission or less than 48 h after admission. Hospital-acquired infection was defined as culture-positive samples collected from patients who had been admitted to the hospital at least 48 h before sample collection, or had been discharged from hospital within two weeks of the present admission or had been born in KCH and admitted directly to the neonatal unit.

### 2.2 Antimicrobial resistance testing

Antimicrobial susceptibility was determined by a broth microdilution minimum inhibitory concentration (MIC) method (AutoScan, Siemens, Germany) in accordance with the Clinical Laboratory Standards Institute (CLSI) guidelines (Andrews, 2001). Extended Spectrum β-Lactamase (ESBL) testing was performed using the double disk diffusion method following the CLSI guidelines (2013). Multi-drug resistance (MDR) was defined as non-susceptibility to at least one agent in three or more antimicrobial classes (Magiorakos et al., 2012), excluding ampicillin, as *K. pneumoniae* is intrinsically resistant to ampicillin.

### 2.3 DNA extraction and whole genome sequencing

Overnight cultures of *K. pneumoniae* isolates grown on Cysteine Lactose Electrolyte-Deficient (CLED) agar were harvested and emulsified in 500 μL 1% Tris EDTA to form a 0.5 McFarland solution. Samples were centrifuged at 5340 Rcf for 10 min. The supernatants were discarded while the pellets were extracted using the QIAamp^®^ DNA mini kit (Qiagen, Germany) according to the manufacturer’s instructions for isolation of genomic DNA from bacterial cultures. DNA was eluted in 60 μL and quantified using an Invitrogen Qubit^®^ 2.0 Fluorometer (Life Technologies, USA). The DNA for 190 isolates was subjected to paired-end whole genome sequencing on an Illumina HiSeq2000 Platform at the Wellcome Trust Sanger Institute (Hinxton, United Kingdom) to generate 100 bp paired-end reads. DNA for eight isolates were sequenced on Illumina MiSeq to generate 150 bp paired-end reads (Quail et al., 2011).

### 2.4 Mapping and assembly

The sequence reads for each isolate were aligned to *K. pneumoniae* MGH 78578 (RefSeq: GCF_000016305.1) genome using a pipeline developed in-house. Reads were aligned using SMALT (http://www.sanger.ac.uk/resources/software/smalt/). The isolates were assembled using an in-house pipeline that included Velvet v1.2.09 (Zerbino and Birney, 2008), SSPACE (Boetzer et al., 2011) and GapFiller (Boetzer and Pirovano, 2012). The reads were aligned back to the improved assembly using SMALT and a set of statistics was produced for assessing the QC of the assembly. De novo assemblies with either N50 <30,000 bp or total assembled nucleotides <4 Mb were excluded from further analyses. The method is described in more detail in the supplementary section. Sequence data are available in the European Nucleotide Archive database (accession no.: ERP002304).

### 2.5 Genome annotation and the core genome

Assembled contigs were annotated using Prokka (Seemann, 2014). The predicted genes were then annotated with the aid of a *Klebsiella* genus-specific database resulting in a GFF file containing an annotated *de novo* draft assembly.

The core genes were determined from the annotated *de novo* assemblies: Predicted coding regions were extracted and converted to protein sequences. Sequences where more than 5% of nucleotides were unknown or which were less than 120 nucleotides in length were excluded from further analysis. Sequences without a start or stop codon were filtered out. CD-hit was used to iteratively perform a first pass clustering. The protein sequences were then clustered beginning with a sequence identity of 100% and a matching length of 100%. If a sequence was found in every isolate, it was said to be a conserved gene and the cluster added to the final results. All of these sequences were then removed and not considered for blast analysis. CD-hit was repeated again with a lower threshold, reducing by 0.1% down to 98%, with conserved clusters removed at each stage. The clusters were labeled with the most commonly occurring gene names assigned to the sequences in the cluster. If there was no annotated gene name, a unique identifier was generated. The functional annotation was also recorded for each cluster.

### 2.6 MLST

For Multi-Locus Sequence Typing (MLST) all genomes were mapped against the seven housekeeping genes, *gapA, infB, mdh, pgi, phoE, rpoB* and *tonB,* that make up the *K. pneumoniae* MLST scheme (Brisse et al., 2009, Diancourt et al., 2005). The sequence types (ST) for each isolate were determined using the scheme for *K. pneumoniae*. Novel alleles and profiles were submitted to the MLST database (http://bigsdb.web.pasteur.fr/klebsiella/klebsiella.html). Clonal complexes (CC), defined as a group of profiles that differ by no more than one gene from at least one other profile of the group, were determined from the MLST profiles of isolates with known alleles. The CC nomenclature used is specific to this study.

### 2.7 Phylogeny and divergence

A phylogeny was drawn for *K. pneumoniae* using RAxML v7.8.6 (Stamatakis, 2006) to estimate the trees (100 bootstrap replicates) for the core genome and the General Time Reversal model with gamma correction.

Average divergence within a lineage was calculated on the core genes by comparing the number of base differences per site from averaging over all sequence pairs within each lineage. Gaps were disregarded from the calculation. Divergence between lineages was calculated using the same method. MEGA v6.06 was used for the calculations.

### 2.8 Detection of antimicrobial resistance genes

The resfinder database of bacterial antimicrobial genes (Zankari et al., 2012) was manually curated in-house to remove redundancy and partial genes, creating the resistome. Ariba (https://github.com/sanger-pathogens/ariba/wiki) was used to search *de novo* assembled contigs against the non-redundant resistome, which comprised of 1777 resistance genes. Search hits with >90% nucleotide identity over >80% of the resistance gene, were considered a match.

### 2.9 Identification of NDM-1 plasmid

Reads from all isolates were mapped against the pNDM-MAR sequence (GenBank accession: JN420336.1) and that of pNDM-KN (GenBank accession: JN157804) using mapping methods described above. Contigs from the *de novo* assemblies of isolates that mapped with read coverage of ≥6 reads over 70% or more of the plasmid sequence were searched against the plasmid sequence using MUMMER v3.1. A contig was considered to be well mapped to the plasmid if at least 80% of the contig length aligned to the plasmid with a minimum of 90% nucleotide identity. We considered an isolate with coverage of at least 60% to the plasmid to strongly suggest the presence of the plasmid.

## 3 Results

### 3.1 Antimicrobial resistance profile of *K. pneumoniae* from KCH

A total of 52,610 paediatric (0–14 years) and 16,076 adult patients were admitted to KCH in the study periods of 2001–2011 and 2007–2011, respectively. Of these, 92% (48,446) paediatric and 54% (8676) of adult patients were eligible for blood culture of which 97% (46,791) paediatric and 64% (5594) of adult patients consented to blood tests. Blood samples were collected from 97% (45,177) paediatric and 94% (5239) of adult consenting patients. Of these, *K. pneumoniae* was isolated from cultures of blood or CSF from 197 (0.4%) children and 24 (0.4%) adults and stored at −80 C. One hundred and seventy four (88%) isolates from children and all the isolates from adults were successfully located and subcultured and subsequently sequenced. The sample set consisted of near-equal proportions of hospital-acquired infections (101/198, 51%) and community-acquired infections (97/198, 49%).

**Fig. 1:**
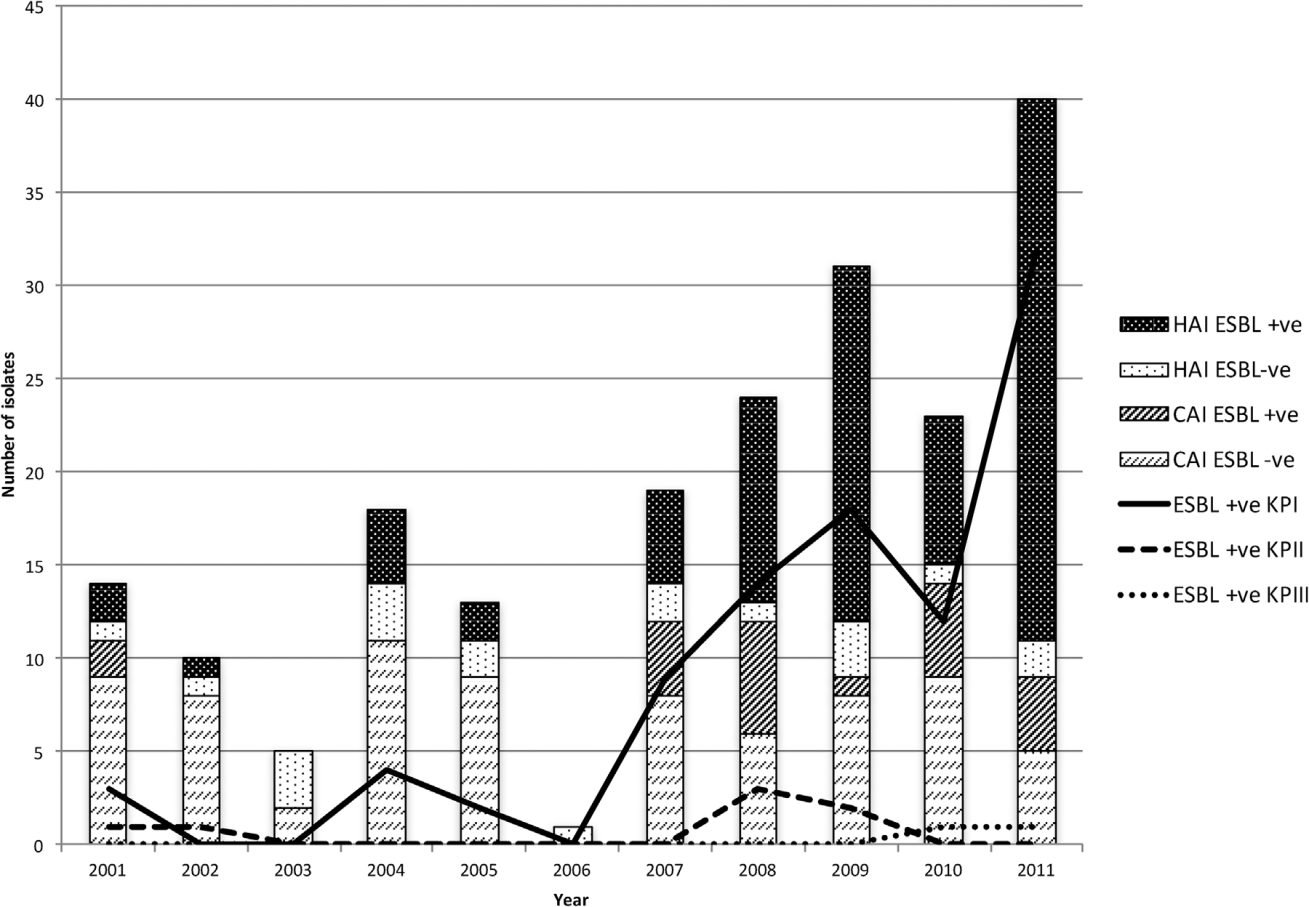
Expansion with time of ESBL-positive *K. pneumoniae* in hospital-acquired infections and the contribution of KPI lineage in this expansion.

The antibiotic susceptibility profile for all isolates is summarised in Table 1. Resistance to gentamicin (and thus to the empiric therapy of ampicillin plus gentamicin for suspected paediatric bacterial infection) was seen to increase significantly (p < 0.001) from 6/14 (43%) in 2001 to 33/40 (83%) in 2011. Gentamicin resistance was observed in 88% (89/101) of isolates from hospital-acquired infections and 37% (36/97) of isolates from community-acquired infections (p < 0.001). ESBL-carrying isolates were observed in 79% (81/103) of hospital-acquired infections and 23% (22/103) of community-acquired infections (p < 0.001). Fig. 1 shows the breakdown of *K. pneumoniae* isolates per year by acquisition status and the proportion of isolates that were phenotypically ESBL-positive. These data show that not only did the proportion of hospital-acquired *K. pneumoniae* increase between 2001 and 2011 but there was an overall increase in the number of ESBL-carrying *K. pneumoniae* isolates. For both community-acquired infections (p = 0.003) and hospital-acquired infections (p < 0.001), the proportion of ESBL-positive isolates increased from 18% (2/11) and 67% (2/3), respectively, in 2001 to 44% (4/9) and 94% (29/31) in 2011. Although no isolates were found to be phenotypically resistant to carbapenems, 79% (N = 159) were multi-drug resistant (MDR), conferring resistance to between three and 11 agents (excluding penicillins) of the 15 antimicrobial categories tested. MDR was observed in 94% (95/101) of isolates of hospital-acquired infections and 63% (61/97) of isolates of community-acquired infections (p < 0.0001).

**Table 1:** Antimicrobial susceptibility testing of 198 *K. pneumoniae* invasive isolates. Susceptible (S), Non-susceptible (NS) and Extended Spectrum Beta-lactamase (ESBL) phenotypes are reported.

	Hospital-acquired (N = 101)		Community-acquired (N = 97)	
Antibiotic	S	NS (ESBL+^b^)	S	NS (ESBL+^b^)
Tetracycline	29	72	44	53
Trimeth/Sulfa^a^	10	91	33	64
Amikacin	94	7	97	0
Gentamicin	12	89	61	36
Ampicillin	0	101	0	97
Cefotaxime/Ceftriaxone	19	81 (79^b^)	68	29 (22^b^)
Ceftazidime	19	82 (79^b^)	68	29 (22^b^)
Imipenem	101	0	97	0
Ciprofloxacin	86	15	84	13

a Trimethoprim-sulfamethoxazole, also known as co-trimoxazole.

b Number of isolates that were non-susceptible to Cefotaxime/Ceftriaxone or Ceftazidime and were ESBL-positive.

### 3.2 Population structure by MLST and whole genome sequences

**Fig. 2:**
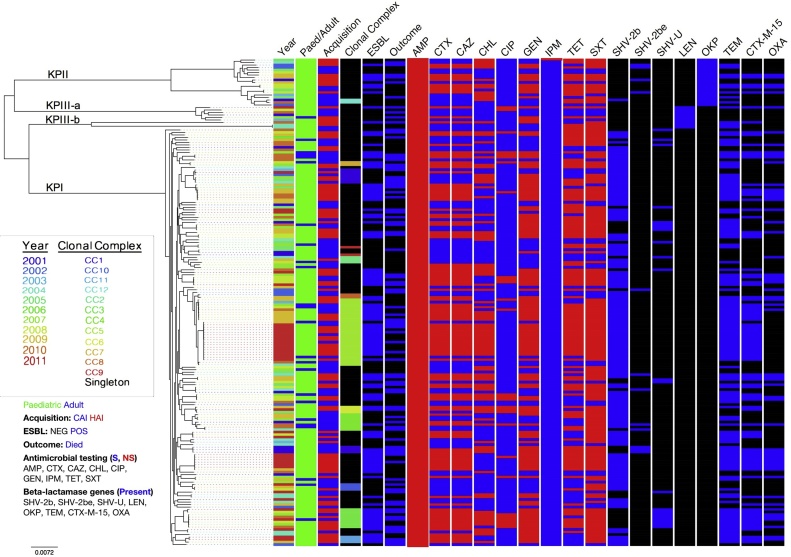
Phylogeny of invasive *K. pneumoniae* isolates from Kilifi. Maximum likelihood tree was constructed on the 1874 core genes. Metadata for each isolate are shown as coloured boxes. Paediatric samples are shown as green boxes, community-acquired infections (CAI) are shown as red boxes, while adult samples and hospital-acquired samples (HAI) are shown as blue boxes. Antimicrobial susceptibility testing for Ampicillin (AMP), Cefotaxime (CTX), Ceftazidime (CAZ), Chloramphenicol (CHL), Ciprofloxacin (CIP), Gentamicin (GEN), Imipenem (IPM), Tetracycline (TET) and Trimethoprim-sulfamethoxazole (SXT) is shown as resistant (red) and non-resistant (blue) phenotype. ESBL-positive samples are indicated with a blue box. β-lactamase genes (SHV, LEN, OKP, TEM, CTX-M-15, OXA) are grouped by type (blue if present). *bla*_SHV_ β-lactamases are grouped into three phenotype classes – 2b, 2be and unknown (U). For the β-lactamase genes, ESBL and outcome (died) columns black boxes indicate absence of the respective phenotype in the sample. The inset shows the colour keys for the year the sample was isolated in and the clonal complex it belonged to. (For interpretation of the references to colour in this figure legend, the reader is referred to the web version of this article.)

Whole genome sequences for the 198 *K. pneumoniae* isolates were obtained. Three isolates were excluded from further analyses due to having genome assemblies with greater than 2000 contigs and an average contig length of <3000 bp. The assemblies of the remaining 195 isolates had a median of 85 contigs (range: 22–2895) and N50 of 282,488 (range: 32,354–747,095). By MLST analysis, 58 distinct STs were represented in 130 isolates; 34 STs in 71 isolates from hospital-acquired infections and 36 STs in 59 isolates from community-acquired infections. Novel STs were found in 28 isolates and 52 novel alleles were found in 37 isolates. Therefore, a total of 138 distinct STs were distinguished. The isolates grouped into 12 clonal complexes (CC), comprising a maximum of three STs and 88 singletons (Table S1). A majority of the CC had approximately equal proportions of community and hospital-acquired isolates, however three were comprised of only community-acquired isolates and one of solely hospital-acquired isolates. STs of half the isolates occurred only once in the decade. However, STs such as ST15 (CC3) and ST17 (CC5) were seen to recur almost every year since their emergence (Fig. 2).

Together with the large number of distinct STs in the sample set, considerable sequence diversity was also observed. Loci *gapA*, *infB*, *mdh*, *pgi*, *phoE*, *rpoB* and *tonB* had 24 (5.3%), 29 (9.1%), 70 (14.7%), 61 (14.1%), 43 (10.2%), 52 (10.4%), 93 (24%) polymorphic sites, respectively. Three insertion/deletion (indels) events at the *tonB* locus (4, 2 and 2 codons, respectively) were observed. Excluding these indels 372 of 3012 bp (12.4%) were polymorphic when the seven loci were concatenated. In isolates from hospital-acquired infections and community-acquired infections 335 (11.2%, excluding gaps) and 326 (10.8%, excluding gaps) sites were polymorphic, respectively. This high genetic diversity observed in MLST groupings was in complete agreement with clustering defined by core genes obtained from whole genome sequencing. The core gene set of 1874 genes was derived from a comparison of all of the coding sequences found in the whole genomes of the 195 isolates. Within 1.2 Mb comprising the core gene set 149,545 (14%) nucleotide sites varied. In addition, 17,439 unique genes were identified among the 195 isolates.

Molecular typing using *gyr*A and *par*C genes has identified three phylogenetic lineages of *K. pneumoniae* globally to date (Brisse and Verhoef, 2001, Holt et al., 2015). They are KPI, KPII and KPIII. Two subgroups within the KPII lineage have also been defined (Fevre et al., 2005).

A maximum likelihood phylogenetic analysis showed that the Kilifi isolates fell in four lineages/sublineages, three of which are globally known (Fig. 2, Fig. S1). In total, 166, 19 and nine isolates from the sample set fell into the known lineages, KpI, KpII and KpIII, respectively. The fourth lineage identified in this study comprised three isolates and is described in more detail in a subsequent section. All four lineages included isolates from both hospital-acquired infections and community-acquired infections. An expansion was noticed in the KPI lineage (p = 0.0048) where the number of isolates per year increased from 11 in 2001 to 39 isolates in 2011 (Fig. 1). There was also an increase per year in the proportion of isolates in the KPI lineage causing hospital-acquired infections (p < 0.001), from ∼20% (2/11) to ∼80% (30/39). The number of ESBL–positive isolates also increased from ∼10% to ∼93% in the same time period in the KPI lineage (p < 0.001). No temporal trends could be observed for the other lineages due to the small number of isolates.

### 3.3 Analysis of outbreaks in 2009 and 2011

The phylogenetic analyses also confirmed outbreaks that had occurred at KCH: In 2009, sequential infections caused by a multidrug resistant ST17 (CC5) ESBL-positive strain affected four infants within a span of one month in the neonatal unit, including two fatalities. Isolates from three of the patients were from hospital-acquired infections by formal definition, while the fourth patient was born in the hospital prior to being admitted to the ward. The core genome of all four isolates was identical (Fig. 1). A second outbreak by an isolate from a closely related lineage, also of ST17 but differing by 1689 (0.1%) point mutations in its core genes compared to the 2009 isolates, was seen in 2011. On this occasion affecting 14 neonates in the span of 36 days; 12 isolates were from hospital-acquired infections and two were from community-acquired infections. Nearly half (n = 6) of the 14 cases died. With the exception of two, all invasive *K. pneumoniae* infections within this period were ST17. The phenotypic and genotypic profiles of antimicrobial resistance were identical in all 18 ST17 isolates identified in 2009 and 2011. Sporadic community-acquired infections caused by isolates belonging to ST17 in both newborns and adults were seen on six additional occasions between 2004 and 2011, where four variants had lost the ESBL phenotype.

A series of hospital-acquired infections caused by an MDR isolate belonging to ST70 (singleton) affected six neonates within 15 days in 2011. This ST was also seen on one previous occasion in a hospital-acquired infection in 2009, sharing the same phenotypic and genotypic antimicrobial resistance profiles, and having a near-identical core genome content (99.99% nucleotide identity) to the 2011 isolates.

### 3.4 Genetic determinants conferring resistance to β-lactams

Phenotypic antimicrobial testing revealed that there were 8 classes of antimicrobial resistance genes present in the *K. pneumoniae* isolates taken from KCH (Table 1). Genes were found conferring resistance to penicillins, aminoglycosides, 2nd generation cephalosporins, chloramphenicol, quinolones, fosfomycin, macrolides, sulfonamides and tetracycline (Table S2). At least one copy of the chromosomal ß-lactamase genes, *bla_SHV_*, *bla_LEN_* and *bla_OKP_*, conferring intrinsic resistance to ampicillin was present in all isolates. Only one isolate was found not to carry any other resistance genes, consistent with its phenotypic antimicrobial resistance (AMR) profile, that of a “wild-type” *K. pneumoniae*.

The phylogeny of *K. pneumoniae* lineages KPI, KPII and KPIII has been shown to correspond to that of its innate chromosomal β-lactamase genes *bla*_SHV_, *bla*_OKP_ and *bla*_LEN,_ respectively (Brisse and Verhoef, 2001, Haeggman et al., 2004). These chromosomal β-lactamase variants do not confer resistance to extended spectrum β-lactams. The relationship between lineage and chromosomal β-lactamase variant was also observed in our dataset. All isolates in the KPI lineage (N = 166) carried the chromosomal *bla*_SHV_ gene. In total, 176 *bla*_SHV_ genes that were either chromosomal or plasmid associated, were identified in 171 isolates, from KPI and KPII lineages. No *bla*_SHV_ genes were found in the KPIII lineage. A high degree of variation in the *bla*_SHV_ genes was observed. Overall, 26 enzyme types of *bla*_SHV_ genes were identified (Table S3). Most of these belonged to the 2b group of the Bush and Jacoby functional classification scheme (Bush and Jacoby, 2010) denoting them as encoding penicillinases. Three enzyme types (SHV-2A, SHV-12, SHV-27) belonging to the 2be group, which are ESBLs that confer resistance to extended spectrum cephalosporins, were identified in nine isolates; five were in the KPI lineage while four were in the KPII lineage. For isolates in the KPI lineage genes within ∼14 kb region flanking the *bla*_SHV_ gene were compared for synteny with the chromosome of MGH 78578 (Fig. S3). In all the five isolates the entire ∼14 kb locus, from the chromosomal ygb genes to the lac operon were present and their order maintained. In addition, the latter region was assembled into one contig in all five isolates. Furthermore, copies of intact or truncated transposons were not found in any of the contigs in which this locus was assembled. The above points strongly support the chromosomal origin of the contigs. In three of these isolates multiple copies of the *bla*_SHV_ gene, accompanied by flanking genes, some truncated, were located at the same locus. A full-length copy of *bla*_SHV_ genes belonging to the 2be group was also present at the same locus. In the remaining three isolates from the KPI lineage a 2be *bla*_SHV_ gene had replaced the 2b variant. The *bla*_SHV_ group 2be genes to date have largely been described as plasmid-encoded genes; they have not been identified as chromosomally encoded in *K. pneumoniae*.

The mean pairwise nucleotide divergence as estimated using the core genes was 0.4% and 1.4% within KPI and KPII lineages, respectively. Within the KPII lineage, Kilifi isolates separated into two known subgroups distinguished by the variant of chromosomal *bla*_OKP_ β-lactamase gene, *bla*_OKP-A_ or *bla*_OKP-B_, they carried (Fevre et al., 2005). The nine isolates within the KPIII lineage all carried the chromosomal *bla*_LEN_ gene. Based on their core genes they clustered into two subgroups – KPIII-a and KPIII-b (Fig. S1). The mean pairwise nucleotide divergence within the sequences in KPIII-a and KPIII-b was 0.5% and 2.6%, respectively. Even though they shared the same class of chromosomal β-lactamase gene as KPIII-a, isolates in KPIII-b were genetically closer to KPI (mean pairwise nucleotide divergence = 4.4%) than they were to KPIII-a (mean pairwise nucleotide divergence = 4.6%) or to KPII (mean pairwise nucleotide divergence = 5.2%) suggesting that KPIII-b might share a nearest common ancestor with KPI rather than with KPIII-a. Phylogeny of the *bla*_LEN_ genes of the nine KPIII isolates correlated with that of the core genes (Fig. S2).

In addition to *bla*_SHV_, seven classes of β-lactamase genes often carried on a plasmid were also found in these isolates. These were *bla*_CTX-M_, *bla_OXA_*, *bla*_TEM_, *bla*_DHA1_, *bla*_CARB_, *bla*_CMY_ and *bla_S_*_CO_. Of the clinically significant β-lactamases *bla*_TEM-1_, an enzyme that can hydrolyze benzylpenicillin and early cephalosporins, was present in 134 isolates (68%). Only one variant of the *bla*_TEM-1_ gene was found. A *bla*_CTX-M-15_ ESBL encoding gene was present in 92 (47%) isolates and all but one of the isolates carrying a *bla*_CTX-M-15_ gene had an ESBL phenotype. Four types of *bla*_OXA_ genes were found in 61 isolates. Two *bla*_OXA_ variants, OXA-1 and OXA-10, belong to the 2d phenotypic group (increased hydrolysis of oxacillin), while two variants, OXA-89 and OXA-58 belong to the 2df group that has oxacillinase as well as some weak carbapenemase activity.

### 3.5 Identification of a pNDM-mar-like plasmid backbone

**Fig. 3:**
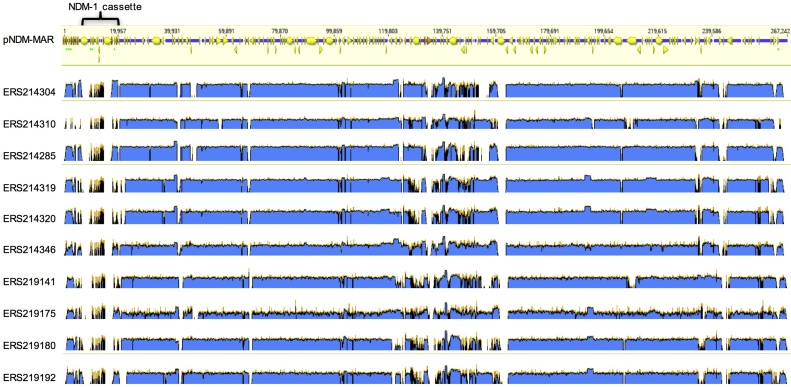
Coverage area plot of ten invasive *K. pneumoniae* isolates showing reads mapped to pNDM-MAR (GenBank accession: JN420336.1). In these isolates the AMR genes are present on the same contig and are flanked by inverted repeats of IS*26*. Gene boundaries in pNDM-MAR are shown as yellow arrows. Antimicrobial resistance genes are marked with green dots underneath the gene. (For interpretation of the references to colour in this figure legend, the reader is referred to the web version of this article.)

Of the 195 isolates sequenced, 25 isolates had reads that mapped to between 72%–88% of the length of the pNDM-MAR plasmid known to carry the metallo β-lactamase gene *ndm-1*. Consistent with this the IncHIB-like and IncFIB- like plasmid replication genes, characteristic of pNDM-MAR, were present in all 25 of these isolates and absent from all other isolates sequenced here. It is clear from Fig. 3 that based on mapping coverage the region that was poorly represented in the sequence data in all the isolates was the ∼16 kb region between the IS26 insertion element that normally brackets the *ndm*-1 cassette from genes *ndm*-1 to *yhxD*, and includes the mercury resistance operon and several transposase-encoding regions. AMR genes *catB*, *bla*_OXA-1_ and *aac(6′)-Ib* are located in tandem on pNDM-MAR, supposedly co-mobilized by IS*26*. In ten isolates these AMR genes were located on the same contig and they were flanked by inverted repeats of IS*26*. The *bla*_CTX-M-15_ gene in pNDM-MAR is flanked by mobile genetic elements (MGEs) IS*Ecp1* and Tn*3.* Nine out of ten isolates carrying the *catB*-*bla*_OXA-1_-*aac(6′)-Ib* cassette also carried *bla*_CTX-M-15_. In all nine isolates *bla*_CTX-M-15_ was flanked by a Tn3 transposable element. Although the plasmid appeared to be present, none of the isolates carried the *ndm-1* gene itself. The plasmid was detected in multiple STs including ST15 (CC3), ST54 (singleton), and 15 other known and 6 novel ST’s. These included isolates from lineages KPI, KPII and KPIII (data not shown).

## 4 Discussion

In Kilifi, as in the rest of the world, multidrug resistant Gram-negative enteric bacteria are of mounting concern. We found dramatically increasing numbers of *K. pneumoniae* bloodstream infection at Kilifi County Hospital over a decade, with a concomitant increase in antibiotic resistance. This increase occurred in parallel with an increase in maternity services so that more babies were being born at KCH and unwell neonates were increasingly referred to the paediatric ward for investigation with blood cultures. By 2011, 94% of hospital-acquired and 44% of community-acquired *K. pneumoniae* bloodstream infections were ESBL-positive, resistant to both first-line and second-line empiric antibiotic treatment. Outbreaks of ESBL-carrying hospital-acquired *K. pneumoniae* infections, suspected clinically, have been confirmed genomically. We have demonstrated high genetic diversity of *K. pneumoniae* invasive isolates in Kilifi, mirroring the high global diversity of this successful pathogen.

Well-equipped microbiology facilities are not commonly found at public hospitals throughout sub-Saharan Africa, so there is a paucity of data on the spread of multidrug resistant Gram-negative pathogens in the region, despite increasing global anxiety. Previous reports of widespread resistance to common first line agents such as ampicillin, trimethoprim-sulfamethoxazole and gentamicin led some hospitals to introduce third generation cephalosporins such as ceftriaxone as second or even first-line empiric antibiotic regimens for suspected infection. We speculate that this approach was probably taking place at the same time as ESBL-carrying Gram-negative bacteria like *K. pneumoniae* were getting a foothold in the region; largely undetected due to a lack of microbiology laboratory capacity. The resulting selection pressure may have increased the generation and spread of ESBL-positive pathogens resistant to ceftriaxone. Our data suggest that multidrug resistance, to ampicillin, gentamicin, tetracycline, trimethoprim-sulfamethoxazole and ceftriaxone, is common among bloodstream isolates of *K. pneumoniae* in Kilifi. Isolates remained relatively susceptible to ciprofloxacin and amikacin; while not ideal treatment and not without potential problems in children, this was fortunate since carbapenems are largely not available at KCH and do not feature on the WHO essential medicines list or Kenyan National Formulary.

Our MLST data suggests that *K. pneumoniae* invasive infections in Kilifi cannot be attributed to any one particular ST for either community or hospital-acquired infections. The ubiquitous nature of this pathogen has also been reported in other studies (Holt et al., 2015). Community strains of ESBL-*K. pneumoniae* had similar STs to the hospital-acquired strains raising the possibility that there may be frequent exchange of either bacterial strains or plasmid-borne resistance genes between the community and hospital environments. Although data on over-the-counter antibiotic use in Kilifi is not available we can assume it has increased with the economic development in the county and the increased availability of generic drugs. The rise in ESBL producing strains in the community could be partially attributed to this. It could also be assumed that economic development over time has seen increased travel into and out of Kilifi County, from within Kenya and internationally – for example travel to India where both ESBL-positivity and carbapenem resistance is common among enteric bacteria carried in the gut.

Carbapenem resistance due to plasmid-borne metallo-β-lactamases in *Enterobacteriaceae* is a more recent and even more alarming development in the global antimicrobial resistance crisis than the emergence of ESBLs. Carbapenemase *bla*_NDM-1_ is one of the most widely reported. NDM-1 (New Delhi Metallo-β-lactamase) is a broad spectrum β-lactamase able to hydrolyse all β-lactams, (Yong et al., 2009) first reported from India. It has been found on several plasmids belonging to a range of incompatibility groups (Carattoli, 2013), and in various *Enterobacteriaceae*. Although we had not phenotypically detected resistance to carbapenems in Kilifi, carbapenem-resistance due to *bla*_NDM-1_ has been reported elsewhere in Kenya (Poirel et al., 2011b). Introduction of carbapenem use in the context of pre-existing *bla*_NDM-1_-carrying plasmids could result in the emergence of isolates resistant to all frontline antimicrobials, as has been seen elsewhere (Chung The et al., 2015). What is more, our sequence data revealed that twenty-five of the isolates showed high nucleotide identity to pNDM-MAR. pNDM-MAR is an IncHI-FI1B plasmid first identified from ST15 *K. pneumoniae* isolates in North Africa and known to carry *bla*_NDM-1_ (Poirel et al., 2011a, Villa et al., 2012). ST15 is a widespread ST (Breurec et al., 2012) that was also found in our samples. Our data showed that the pNDM-MAR plasmids carried by isolates in our collection shared the same genetic architecture as pNDM-MAR, except all lacked the *ndm*-1 cassette. Many of the plasmids had a truncated IS*26* gene which is associated with the transfer of the *ndm*-1 cassette. Importantly IS*26* can excise adjacent genes via a *recA* independent mechanism (Chung The et al., 2015; Poirel et al., 2011a; Solé et al., 2011) and is known to introduce a high level of instability of part of the pNDM-MAR plasmid. More specifically this instability frequently results in the loss of the *ndm*-1 gene itself and therefore loss of resistance to carbapenem antimicrobials. We hypothesise that in the absence of carbapenem usage in KCH, the gene encoding the NDM carbapenemase could have been lost from this plasmid. The fact that pNDM-MAR is carried by a large number of STs, may suggest that this plasmid has entered the general population and is maintained by ESBL resistance. Since the *ndm*-1 gene is lost rapidly in the absence of selection pressure, it is possible that a comparatively rare plasmid carrying the *ndm-1* gene is leaching into the general population from other regions or from private clinics in Kenya that do use carbapenems. Upon entering the general population where there is no carbapenem-use this gene is lost. More recently, a few cases of phenotypically carbapenem-resistant *K. pneumoniae* have been reported in KCH (personal communication). The increasingly limited treatment options for MDR *K. pneumoniae* and the availability of off-patent imipenem, may subsequently result in the introduction of carbapenems at KCH. When this occurs the introduction of such a plasmid, bearing NDM, may quickly trigger the selection and spread of carbapenem resistance (Huang et al., 2013).

## Funding

This work was supported by the Wellcome Trust [grant number WT098051]. CJB was supported by the Medical Research Council, [grant number G1100100/1]. JAS was supported by a Fellowship from the Wellcome Trust [098532].

## Conflict of interest

The authors declare that they have no competing interests.

## Nucleotide sequence accession number

WGS data is available at the European Nucleotide Archive (ENA) (http://www.ebi.ac.uk/ena) under the study accession numbers detailed in Table S1.
