# Supplementary material for: Molecular epidemiology of Klebsiella pneumoniae invasive infections over a decade at Kilifi County Hospital in Kenya
Source: Int J Med Microbiol. 2017 Oct;307(7):422–9. doi: 10.1016/j.ijmm.2017.07.006 (PMC5615107; doi:10.1016/j.ijmm.2017.07.006)
Supplement: Supplementary file 1 [file mmc1.docx]

**Table S1.** ST and alleles assigned to MLST loci and their corresponding sequence for each isolate.

**Table S2.** Antimicrobial resistance genes identified in the isolates.

**Table S3**. Alleles identified in 165 full-length *bla*_SHV_ sequences from this study. Only polymorphic residues relative to allele 1 are shown. Where polymorphisms are present the alternate residue is given. Residue positions are relative to *bla*_SHV-1_ (GenBank Accession: AF148850_1). Functional group and known allele names are assigned based on Bush and Jacoby classification found at <http://www.lahey.org/studies/>.
