# Supplementary figures and images for: Molecular epidemiology of Klebsiella pneumoniae invasive infections over a decade at Kilifi County Hospital in Kenya
Source: Int J Med Microbiol. 2017 Oct;307(7):422–9. doi: 10.1016/j.ijmm.2017.07.006 (PMC5615107; doi:10.1016/j.ijmm.2017.07.006)

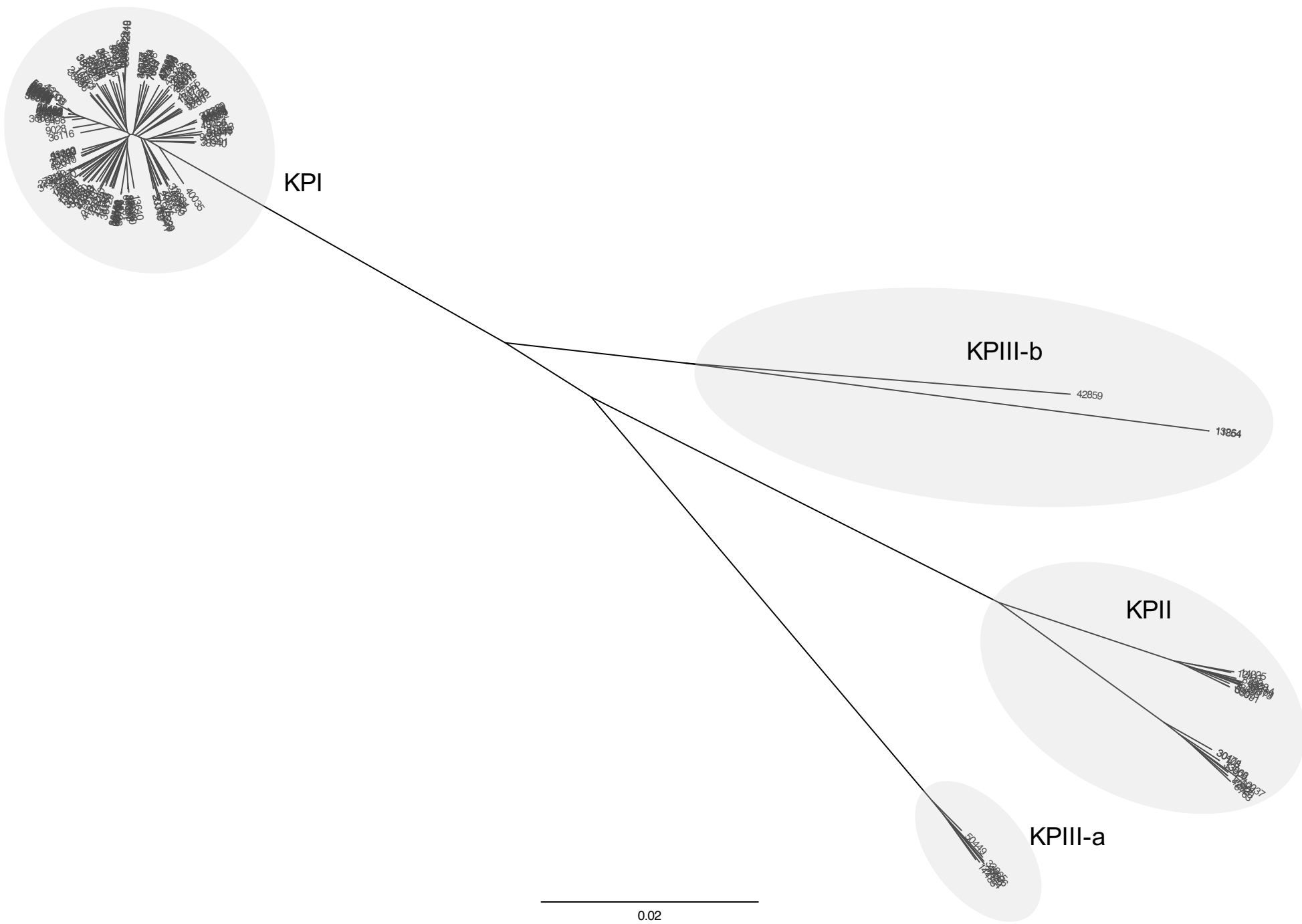

Supplement: Supplementary file 2 [file mmc2.pdf]

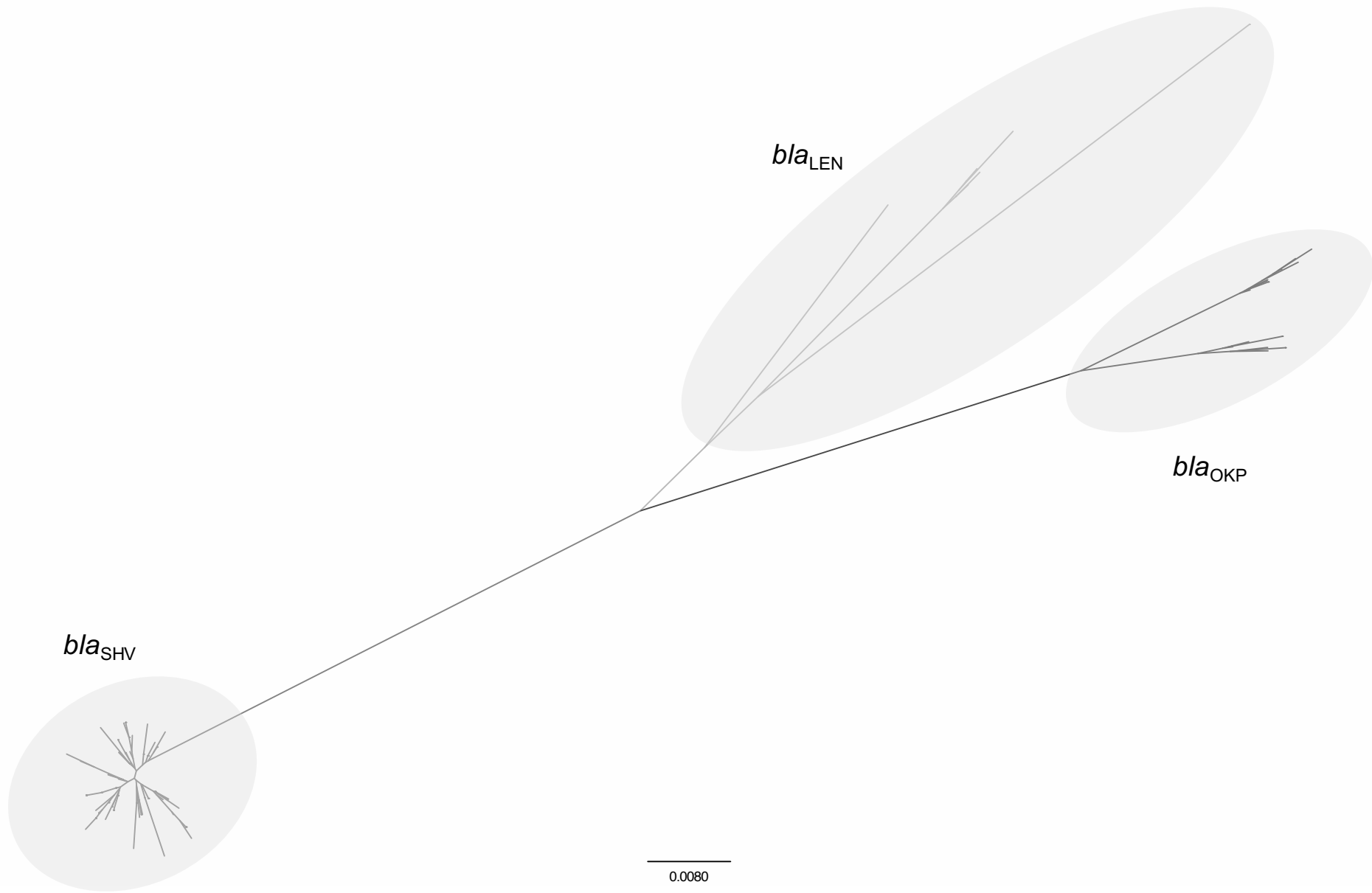

Supplement: Supplementary file 3 [file mmc3.pdf]

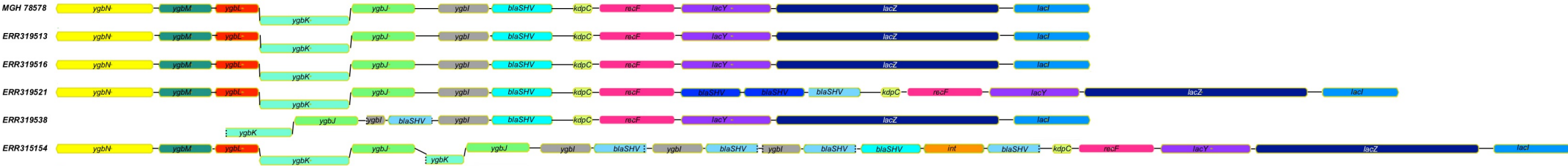

Supplement: Supplementary file 4 [file mmc4.pdf]
