# Supplementary material for: Molecular epidemiology of Klebsiella pneumoniae invasive infections over a decade at Kilifi County Hospital in Kenya
Source: Int J Med Microbiol. 2017 Oct;307(7):422–9. doi: 10.1016/j.ijmm.2017.07.006 (PMC5615107; doi:10.1016/j.ijmm.2017.07.006)
Supplement: Supplementary file 5 [file mmc5.pdf]

Supplemental Methods

Mapping and Assembly

The sequence reads for each isolate were mapped to *K. pneumoniae* MGH 78578 genome (RefSeq: GCF_000016305.1) using a pipeline described in (38). For each strain, the raw read pairs were split into smaller chunks. This reduces the overall memory usage, and allows for the reads to be aligned using more than one CPU. The reads were then aligned using SMALT, a hashing based aligner. The aligned reads and unmapped reads were merged into a single BAM file. Picard (http://picard.sourceforge.net) was used to identify and flag optical duplicates, which reduces the effects of PCR bias.

The isolates were assembled using a pipeline developed in-house. For each strain, Velvet v1.2.09 (17) was used to create multiple assemblies by varying the kmer size between 66 % and 90 % of the read length. From these assemblies, the one with the best N50 was chosen and contigs which were shorter than the insert size length were removed. An assembly improvement step was then run on the chosen assembly. The contigs of the assembly were scaffolded by iteratively running SSPACE (18). Then gaps identified as 1 or more N's, were targeted for closure by running 120 iterations of GapFiller (19). Finally, the reads were aligned back to the improved assembly using SMALT (http://www.sanger.ac.uk/resources/software/smalt/) and a set of statistics was produced for assessing the QC of the assembly. *De novo* assemblies with N50 < 30,000 and assembled nucleotides < 4 Mb were excluded from further analyses.

Genome annotation and core genome

Assembled contigs were annotated using Prokka (20) which predicts proteins, RNA structures, tRNA, rRNA and tmRNA. The predicted genes were then annotated with data pulled from a number of databases. A *Klebsiella* genus-specific databases, generated by retrieving the annotation for all of the genomes for a genus from RefSeq (39), was used. The protein sequences were then merged using CD-hit (40) to produce a non-redundant blast protein database. Next UniprotKB/SwissProt (41) was consulted. To qualify for inclusion, a protein must not be a "Fragment" entry; and have an evidence level ("PE") of 2 or lower, which corresponds to experimental mRNA or proteomics evidence. The

protein was then looked up against the HMM profiles from Clusters, Conserved domain database, Tigrfams, and Pfam (A). The result was output as a GFF file containing an annotated *de novo* assembly.

The core genes were determined from the annotated de novo assemblies. Predicted coding regions were extracted and converted to protein sequences. Sequences where more than 5 % of nucleotides were unknown or which were less than 120 nucleotides were excluded from further analysis. Sequences without a start or stop codon were filtered out. CD-hit was used to iteratively perform a first pass clustering. The protein sequences were then clustered beginning with a sequence identity of 100 % and a matching length of 100 %. If a sequence was found in every isolate, it was said to be a conserved gene and the cluster added to the final results. All of these sequences were then removed and not considered for blast analysis. CD-hit was repeated again with a lower threshold, reducing by 0.1 % down to 98 %, with conserved clusters removed at each stage. The clusters were labeled with the most commonly occurring gene names assigned to the sequences in the cluster. If there was no annotated gene name, a unique identifier was generated. The functional annotation was also recorded for each cluster.
